# Sex-specific socioeconomic risk factors for spontaneous subarachnoid hemorrhage—a case–control study during the 5 years before ictus

**DOI:** 10.3389/fpubh.2024.1434742

**Published:** 2024-10-16

**Authors:** Elisabeth Ronne-Engström, Emilie Friberg

**Affiliations:** ^1^Neurosurgery, Department of Medical Sciences, Uppsala University, Uppsala, Sweden; ^2^Insurance Medicine, Department of Clinical Neuroscience, Karolinska Institutet, Stockholm, Sweden

**Keywords:** subarachnoid hemorrhage, gender study, socioeconomic, risk profile, stroke

## Abstract

**Background:**

There is a difference in the incidence of spontaneous subarachnoid hemorrhage (SAH) between sexes, with the majority of cases occurring in female patients. Although this phenomenon has been studied from a medical perspective, the reasons for the predominance of female cases are still unclear. Non-medical factors, such as a patient’s socioeconomic situation, can differ between female and male patients, with health implications. The aim of the study was to identify socioeconomic profiles for both sexes that may be vulnerable to developing SAH. This information could potentially be used for active preventive health efforts.

**Methods:**

This study was based on a 7-year consecutive cohort of 890 patients with SAH treated at Uppsala University Hospital, along with a sex- and age-matched 5:1 control group from Statistics Sweden. The collected information included demographic data, income that was analyzed through “earnings” (EAs), which is defined as the sum of income and other economic compensations related to work, and “disposable income” (DI), which is the net amount that an individual can use. Pension and sickness-related absence from work were measured using early pension (EP), old age pension (OAP), sickness absence (SA), and disability pension (DP). Univariate and multivariate analyses were used.

**Results:**

Among the women, the socioeconomic risk profile for SAH included lower education, unemployment, being registered as living single, residing in a sparsely populated municipality, and increased age. For the men, the risk profile included residing in a sparsely populated municipality and changes in civil status. Both women and men with SAH had lower EAs and DI compared to the controls. Notably, a significantly higher proportion of the women with SAH received DP compared to the controls.

**Conclusion:**

Residing in a sparsely populated area was associated with an increased risk for SAH for both women and men. The women with SAH were more economically vulnerable, whereas the men faced a different type of vulnerability related to changes in civil status. We suggest that healthcare organizations use this information to identify individuals at risk and actively implement preventive measures according to stroke guidelines for both groups.

## Introduction

Spontaneous subarachnoid hemorrhage (SAH) is usually caused by a ruptured cerebral aneurysm and is a condition with high morbidity and mortality. SAH accounts for 5 % of stroke incidents and affects a younger population. It is a general observation that both ruptured and unruptured aneurysms are more common in women than in men [see, e.g., (1–3)]. Studies on sex distribution have approached this topic from various medical angles [for references, see (4)]. These studies are based on sex assigned at birth and have concluded that multiple other mechanisms must be involved. Another common observation in the literature is the association between lower socioeconomic conditions and an increased incidence of SAH (5–7). Socioeconomic factors have been described in various ways, such as rates of unemployment and education levels (5), as well as through various national indexes (6, 7). Nevertheless, it seems that regardless of how they are defined, an individual’s socioeconomic situation can affect their vulnerability to various medical conditions. Considering that the majority of SAH patients are women, as well as that women generally have lower incomes and are more economically vulnerable (8), we wanted to explore the socioeconomic situation before the ictus in more detail. The aim of the study was to identify socioeconomic profiles for both sexes that may be vulnerable to developing SAH. This information could potentially be used for active preventive health efforts.

The study included a 7-year consecutive cohort of patients with spontaneous SAH treated at the Department of Neurosurgery, Uppsala University Hospital, and a sex- and age-matched control group drawn from the general population of Sweden. The SAH cases and controls were compared and stratified by sex regarding the demographic and economic situation and pension/sickness-related absence from work five years before the ictus. To the best of our knowledge, this has not been studied previously in a defined patient cohort.

## Materials and methods

### Study design

The project was a case–control study based on a cohort of 890 patients who were treated at the Department of Neurosurgery, Uppsala University Hospital, from 2012 to 2018 for spontaneous SAH. A control group consisting of five individuals per patient (*n* = 4,450 controls) who did not have SAH was generated from the general population by Statistics Sweden (SCB). Statistics Sweden maintains the official Swedish national population statistics. Their database describes the entire Swedish population based on the registration of births, deaths, and dates of immigration and emigration. The data on incomes, employment, and pensions used in our study are generated by the tax system, which covers the entire population aged 18 years or older, regardless of whether individuals are employed or not.

The controls were matched to the cases by age and sex, which are known confounders in socioeconomic comparisons. The cases and controls were of the same age during the same calendar year, allowing for a comparison of the number of sick absences, disability pensions, and the actual levels of income. The study period covered five calendar years before the ictus, (year -5 to year -1), and the calendar year of the ictus or SAH was defined as year 0.

### Data sources

Information on age, sex, country of birth, civil status, family situation, education level, work situation, and type of living area was obtained from the longitudinal database for income and labor market studies (LISA). In the present dataset, sex is defined according to SCB as either female or male. This includes any eventual changes in sex. For analyzing income, we chose “earnings” (EAs), which is defined as the sum of income from employment, business, and other work-related economic compensation, including sickness absence (SA) benefits covered by the employer. Income was also measured as “disposable income” (DI), which is the sum of all earnings from employment and businesses, interest from savings, various tax-generating reimbursements, pensions, and various other benefits (child benefit, housing benefit, etc.) minus taxes, payments on student loans, and child support. DI is the net amount of money that can be used for spending. DI is available for different units, such as families and households. In the present study, we used the individual DI. The population density in the living area was calculated using SCB’s information on the number of inhabitants per square km for each municipality in 2018. Finally, we measured the percentage of individuals who had early pension (EP), old age pension (OAP), sickness absence (SA), or disability pension (DP) each year and defined this as pension or sickness-related absence from work. The gross time periods of registered SA and DP were obtained from The Swedish Social Insurance Agency’s Micro-Data for Analysis of Social Insurance register (MiDAS) (9).

### Demography

The following dichotomies were established: born in a Nordic country (Sweden, Norway, Finland, Denmark, and Iceland) versus non-Nordic country (the rest of the world) and low educational level (≤ 9 years) versus high education level (>9 years including high school, university, or equivalent). The population density of the municipality in which the participants lived in the year before the SAH was calculated and classified as sparse or dense (< or > 100 persons/km^2^). In Sweden, 236 municipalities are classified as sparsely populated and 54 as densely populated according to this definition, representing 51 and 49% of the Swedish population, respectively. Unemployed referred to the situation in the year before the SAH (year -1) and was defined as not having control data filed by the employer/company with the tax authorities. Living single also referred to the situation in year -1 and included the individuals who were registered as living as single in the home, with or without children. Change in civil status referred to any changes during the five study years. Change in the municipality of residence was defined as movements to another municipality during the five study years.

### Income

In total, the study covered 26,700 individual years. Years with no EAs were excluded from the comparison of EA levels. For the women, 36% of the study years lacked EAs, and in 82% of those years, OAP, EP, or DP was provided. For the men, the figures were 30 and 67%, respectively. All years were included in the DI comparisons.

### Pension/sickness-related absence from work life

SA, DP, EP, and OAP existed separately or in various combinations for each individual. We classified this information into three groups to indicate different levels of health problems: “Pension,” which included only EP and/or OAP; “Sickness Absence,” which included all combinations with SA except for DP; and “Disability Pension,” which included all combinations that involved DP.

### Statistical analyses

Three different analyses were performed. First, the study material was described and compared between the case and control groups (Table 1). Second, we analyzed whether having SAH could be predicted by preceding socioeconomic factors. This was performed using general linear/nonlinear modeling of the best subsets of variables according to the Akaike information criterion (10). It was performed separately for the women and men. The dependent variable was case–control (binomial), and all predictor variables in the study, birth country, educational level, employment, population density in the residential municipality, moving to another municipality, living single or not, change in civil status, and age at the time of the ictus, were entered and tested. Third, we compared the levels of EA and DI, as well as the number of SA, between the cases and controls. The data were generally not normally distributed; therefore, non-parametric methods were used for descriptive and analytical statistics. Medians (interquartile range, IQR) were used, and the comparison of values was performed using the Mann–Whitney U test, while distributions were analyzed with the χ^2^ test. A *p*-value of <0.05 was considered significant. Statistical 13, Dell Inc., (Tulsa Oklahoma) was used for the statistical evaluation and graphical presentation.

**Table 1:** This table shows the demographic variables for the four study groups: female SAH cases, female controls, male SAH cases, and male controls.

Demography	Women with SAH	Women in the control group	χ^2^
*N* =	544	2,720	…
Age (median; IQR)	59 (51;68)	59 (51;68)	…
	%	%	…
Born in a Nordic country	90.2	87.5	ns
Lower education level	25.9	19.8	*p* < 0.0015
Unemployed	39.2	33.6	*p* < 0.012
Registered as living single	46.4	43.3	ns
Changed civil status	9.7	10.0	ns
Changed municipality of residence	8.3	6.6	ns
Living in a sparsely populated municipality	75.1	56.7	*p* < 0.00001
	Men with SAH	Men in the control group	χ^2^
*N* =	346	1730	…
Age (median; IQR)	58 (48;66)	58 (48;66)	…
	%	%	
Born in a Nordic country	88.1	87.5	ns
Lower education level	26.2	22.6	ns
Unemployed	26.8	27.3	ns
Registered as living single	40.5	37.7	ns
Changed civil status	11.0	8.5	ns
Changed municipality of residence	7.8	8.1	ns
Living in a sparsely populated municipality	71.1	57.3	*p* < 0.00001

A univariate statistical comparison was performed using *χ*^2^-analysis. The female SAH cases had lower educational level and were more often unemployed compared to the controls. SAH cases of both sexes more likely lived in sparsely populated municipalities compared to the controls.

### Ethical review

The Swedish Ethical Review Authority granted permission for the study.

## Results

### Demography

Table 1 shows the demography and the univariate analyses of the distribution between the SAH cases and controls, stratified by sex. The women who later experienced SAH had a lower educational level and a higher rate of unemployment compared to the controls. Living in a sparsely populated municipality the year before the SAH was more common in the case groups than in the controls, both for women and men.

The results from the multivariate analyses are presented in Table 2. The table shows the three best subsets of the variables that were associated with the later occurrence of SAH. For the women, the combination of living in sparsely populated municipalities, having a lower education, being unemployed, registered as living single, being of older age, and being born outside of the Nordic countries was associated with an increased risk of SAH. For the men, the best three models included the combination of living in a sparsely populated municipality, changing civil status, and living single.

**Table 2:** This table shows the results of testing eight socioeconomic variables using multivariate analysis and model building according to the Akaike information criterion (see methods).

Subset of variables	*p*-value	Relative likelihood
Women		
Residence in a sparsely populated municipality	<0.000001	1
Unemployed		
Older age		
Low Education		
Registered as living single		
Not born in a Nordic country		
Residence in a sparsely populated municipality	<0.000001	0.97
Unemployed		
Older age		
Registered as living single		
Not born in a Nordic country		
Residence in a sparsely populated municipality	<0.000001	0.76
Unemployed		
Older age		
Low Education		
Registered as living single		
Not born in a Nordic country		
Changed municipality or residence		
Men		
Changed civil status	<0.000008	1
Residence in a sparsely populated municipality		
Residence in a sparsely populated municipality	<0.000005	0.83
Residence in a sparsely populated municipality	<0.000014	0.77
Changed civil status		
Registered as living single		

These three models give the best subsets of variables chosen by the algorithm for estimating the likelihood of having a SAH. The relative likelihood is the probability that the model is as good a predictor as the highest scoring model. For women the socioeconomic risk profile includes variables that describe an economic vulnerability e.g. living in sparsely populated municipalities, being unemployed, of older age, registered as living single and born outside Nordic countries. Men had another socioeconomic risk profile including living in sparsely populated municipalities and change of civil status.

### Income

**Figure 1:**
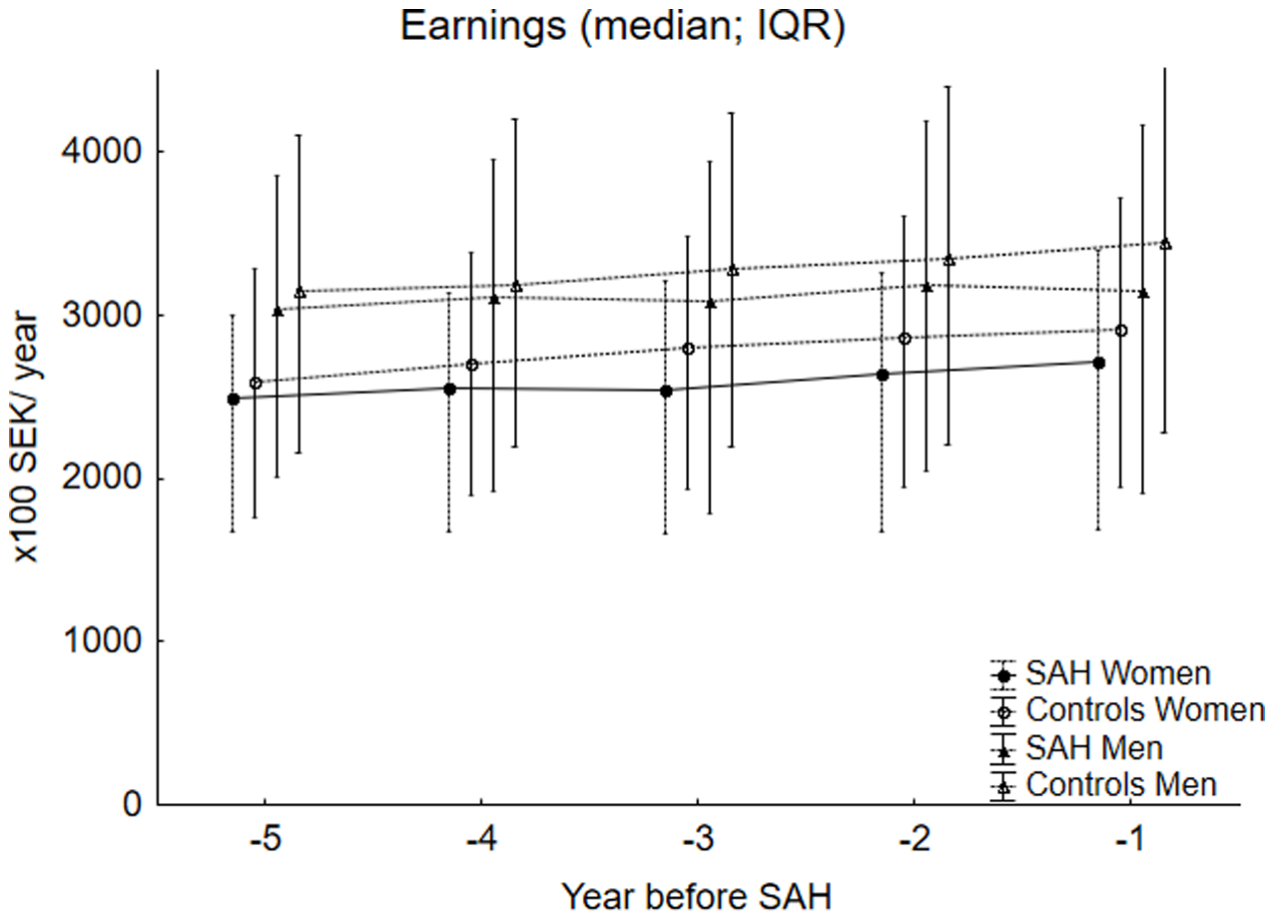
This figure shows the earnings (EA) for the SAH cases stratified by sex (median, IQR). For the women, 36% of the study years lacked EAs, while for the men, it was 30%. The female SAH cases had significantly lower EAs than the controls in all five years preceding the SAH, with *p*-values ranging from *p* < 0.04 to *p* < 0.0004. The men exhibited a similar pattern, with significantly lower EAs for the SAH cases in year -1 and year -3 (*p* < 0.04 and *p* < 0.02, respectively) compared to the controls.

**Figure 2:**
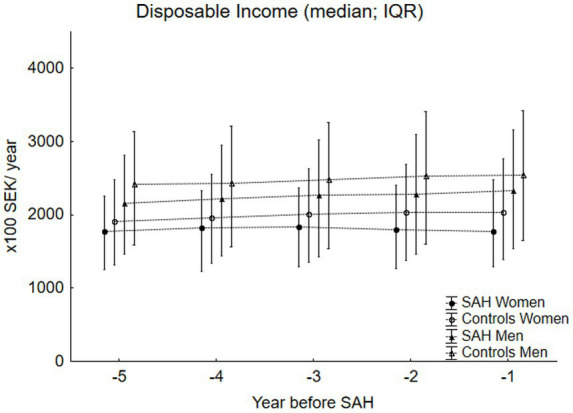
This figure shows the disposable income (DI) (median; IQR) for the SAH cases (*n* = 890) and the controls (*n* = 4,450) stratified by sex. DI was significantly lower for both the female SAH cases (*p*-values ranging from *p* < 0.00001 to *p* < 0.0005) and the male SAH cases (*p*-values ranging from *p* < 0.0002 to *p* < 0.009) in all the five years preceding the SAH, as compared to their controls.

Figure 1 shows the yearly EAs, while Figure 2 shows the yearly DI for the SAH cases and controls, stratified by sex. EAs were significantly lower for the women with SAH than for the controls across all five years, with *p*-values ranging from *p* < 0.04 to *p* < 0.0004. EAs were also lower for the men with SAH across all five years, significantly for year -1 (*p* < 0.04) and year -3 (*p* < 0.02). The DI was significantly lower across all five study years for both women and men with SAH compared to their controls, with *p*-values ranging from *p* < 0.00001 to *p* < 0.0005 for the women and *p* < 0.0002 to *p* < 0.009 for the men.

### Pension/sickness-related absence from work life

**Figure 3:**
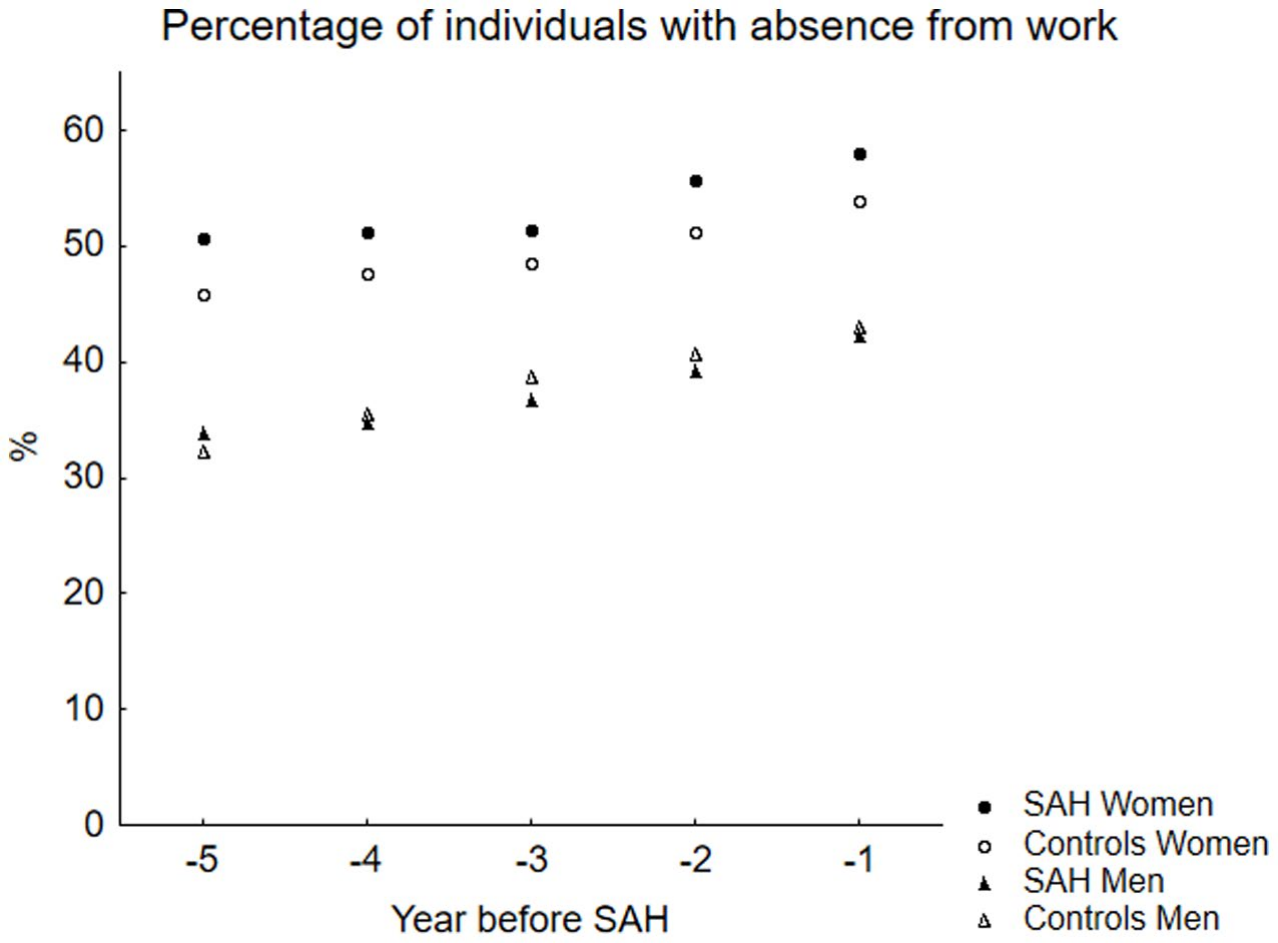
This figure displays the percentage of individuals with work absence related to sickness or pension in the SAH cases and the controls, stratified by sex. There was a higher proportion of the female SAH cases with pension and/or sickness-related work absence compared to the controls, reaching statistical significance in year-5.

Figure 3 shows the percentage of individuals who had any absence from work due to pension, SA, or DP for the four study groups. There was a difference each year in the percentage of individuals with absence from work when comparing the women with SAH and the controls, reaching significance in year -5. In Table 3, the three different groups of absences (pension, SA, or DP) were compared between the SAH groups and the controls. We found that during all five study years, significantly more women who later experienced SAH were on DP compared to the controls. For the men who later experienced SAH, the difference in DP compared to the controls was significant in year -3.

**Table 3:** This table shows the distribution of three types of work absence: sickness absence, old age pension, and disability pension (see methods).

Year before the ictus	Absence	Women			Men		
		SAH	Controls	*p*-value	SAH	Controls	*p*-value
		%	%		%	%	
-5	Pension	23	25	ns	18	19	ns
	SA	11	9	ns	6	6	ns
	DP	17	12	**0.0016**	10	7	ns
−4	Pension	26	27	ns	19	22	ns
	SA	10	10	ns	6	7	ns
	DP	16	11	**0.0038**	9	7	ns
−3	Pension	27	29	ns	23	25	ns
	SA	9	9	ns	5	7	ns
	DP	15	10	**0.001**	9	6	**0.0336**
−2	Pension	30	32	ns	27	29	ns
	SA	12	9	ns	4	6	ns
	DP	14	9	**0.0017**	8	5	ns
−1	Pension	34	36	ns	29	31	ns
	SA	12	10	ns	6	6	ns
	DP	13	9	**0.0037**	8	5	ns

Significantly more women who later experienced SAH received disability pension in all five years before SAH compared to the controls. A similar trend was observed for the men, reaching significance in year -3.

## Discussion

The present study describes the socioeconomic situation before having spontaneous SAH in a patient cohort compared with sex- and age-matched controls. For both women and men, we found differences in the socioeconomic situation between the SAH cases and the controls during the five years preceding the ictus.

Before the ictus, the individuals who experienced SAH more often lived in sparsely populated municipalities, as shown in the multivariate analyses. This was true for both women and men, and the finding is in line with findings from other studies (6). SAH is a type of stroke, and the same preventive measures are important for avoiding SAH, as well as other cerebrovascular and cardiovascular diseases. Lower socioeconomic status has been shown to be associated with higher risks for stroke [for references, see (11)]. It has been suggested that this is due to a relationship between lower socioeconomic status and conventional risk factors for stroke, such as hypertension, smoking, diabetes, and obesity. Primary prevention focuses on promoting a healthy lifestyle regarding diet, exercise, and tobacco use. Secondary prevention involves treating hypertension, diabetes, and other conditions that increase the risk of stroke (12). Central to this preventive work are the primary healthcare centers (PHCCs) with general practitioners and district nurses. Unfortunately, these services have been difficult to maintain in remote areas. There is a global trend, also evident in Sweden, to launch various digital medical services. In a recent study, it was shown that these services were mostly used by younger individuals living in larger cities (13), probably addressing the need for immediate contact regarding various acute conditions, such as infections. It seems unlikely that digital services could replace the PHCC’s prophylactic work; at least this is not been studied yet. Another problem with digital applications is that they are not accessible to a substantial portion of the population, such as older individuals and those with intellectual disabilities.

### Socioeconomic risk profile for women

For the women, we found that models with the combination of living in sparsely populated municipalities, having a lower education, being unemployed, living single, being of older age, and being born outside of the Nordic countries were associated with an increased risk of experiencing SAH. This describes potentially vulnerable individuals, especially from an economic perspective, who may have fewer resources for taking care of their own health. This, together with longer distances to PHCCs, older age, and living single, could lead to a decreased tendency to address health issues through preventive health checkups. This is supported by a study showing that lower access to preventive medicine is due to a higher travel burden (14). We also observed that being born outside the Nordic countries was included in the best subsets of variables. The significance of this finding is not clear. It can be hypothesized that there are differences.

One possible factor contributing to the association between increased SAH risk and economical vulnerability in women is the work situation. Women perform most of the unpaid work, which usually includes caregiving and domestic responsibilities (15). Unpaid work is also a major factor in determining the extent to which women can enter and remain in paid employment (15). Furthermore, a higher burden of unpaid work was associated with a greater depressive symptom trajectory over time (16) for both women and men (17). From this, it can be hypothesized that unpaid workload may contribute to deteriorating health and an increased risk for cardiovascular and cerebrovascular diseases. Our study illustrated that the women with SAH had a significantly worse health status compared to the controls even before the ictus, was as evidenced by a significantly higher frequency of individuals with disability pensions in the SAH group. The relationship between unpaid work and socioeconomic vulnerability in developing cerebrovascular diseases needs further research.

### Socioeconomic risk profile for men

The men who later experienced SAH exhibited a pattern of changing civil status during the five years before the ictus, with living in a sparsely populated area one year before the ictus being more common among them. Mental illness associated with divorce is a known problem in men, and divorce increases the risk of suicide and substance abuse. The loss of social support and connectivity could have negative effects, including a reluctance to seek healthcare for physical and mental issues (18). This situation could be further aggravated by living in a remote area, for the same reasons as noted for the women. The importance of social context was highlighted in a study showing that living single was a risk factor associated with sudden death in SAH (19). They suggested that this may be because a bleeding incident would likely go unwitnessed. It is also possible that this group has a lower tendency to seek health care compared to those with a partner. Change in civil status does not necessarily mean getting a divorce. Other transitions in life that are supposedly positive can also have a negative effect on mental health. Fatherhood and marriages can result in perceived increased demands on different levels, potentially affecting both mental and physical health. Health-related behaviors and beliefs differ between men and women, including the perception that men are independent and robust [for ref. see (20)], which is influenced by societal norms regarding masculinity. Our observations that there were no differences between the men with SAH and the controls regarding registered absence from work support this. However, there was a difference in both EAs and DI between the male SAH cases and the controls, with lower income observed for the men who later experienced SAH. This could indicate economic vulnerability, but it could also signal underlying health problems that lead to less productivity despite being present at work. More studies are needed on the relationship between socioeconomic factors and men’s health.

### Strengths and weaknesses

The strength of this study is that the SAH case group was large, consecutive, and very well-defined clinically, with five age- and sex-matched controls per individual. Therefore, we believe the results are representative. It is important to have a clinically defined group since the ICD-10 coding on a national level is not applied as rigorously as desired, which can affect the quality of register studies. For SAH, a major problem is that many patients with aneurysmal SAH have been coded as SAH with an undefined source of bleeding (I60.9). In addition, traumatic SAH is often coded as spontaneous SAH.

There are some biases that could affect the generalizability of the results. One is that SAH is a type of stroke related to several lifestyle factors, which may depend to some extent on socioeconomic conditions. We do not know how these could change in the future. Tobacco smoking, for example, is one major risk factor for stroke diseases, and its prevalence has decreased significantly in the last decade. It is possible that smoking has been more common in socioeconomically vulnerable situations and that we may not yet have seen the full impact of the decline.

One weakness of this is that it did not cover the actual health conditions and diagnoses underlying SA and DP before the ictus. However, we believe it could be important for political decision-making if risk profiles could be defined based on socioeconomic since they are more available than health data. The medical aspects will be explored in future studies.

## Conclusion

We observed that the individuals who experienced spontaneous SAH displayed different patterns regarding demographics, income, and work situation during the five years preceding the ictus, compared to sex- and age-matched controls. Living in sparsely populated areas was associated with an increased risk for SAH among both women and men. The female SAH cases were more economically vulnerable, with lower income, higher unemployment, and more often living single in sparsely populated areas compared to the controls. For the male SAH cases, a change in civil status was the main risk factor, together with living in sparsely populated areas, indicating a different type of vulnerability. We suggest that healthcare organizations actively work to make it easier for vulnerable groups to access healthcare and receive check-ups. This could be achieved in various ways, such as through home visits. In addition, better information may be needed for individuals born outside the Nordic countries about the preventive healthcare system in Sweden.

## Data Availability

The data analyzed in this study is subject to the following licenses/restrictions: sharing our data is not included in the present ethical review. Requests to access these datasets should be directed to elisabeth.ronne-engström@neuro.uu.se.
